# Effectiveness of Psychotherapy for Internalising Symptoms in Children and Adolescents When Delivered in Routine Settings: A Systematic Review and Meta-analysis

**DOI:** 10.1007/s10567-023-00433-8

**Published:** 2023-04-14

**Authors:** Viviana M. Wuthrich, Dino Zagic, Sophie J. Dickson, Lauren F. McLellan, Jessamine T.-H. Chen, Michael P. Jones, Ronald M. Rapee

**Affiliations:** Centre for Emotional Health, School of Psychological Sciences, Macquarie University, Sydney, 2109 Australia

**Keywords:** Anxiety, Depression, Youth, Effectiveness, Systematic review, Meta-analysis

## Abstract

This systematic review and meta-analysis aimed to examine the effectiveness of psychological interventions for internalising disorders in youth when delivered in routine settings. Secondary aims were to examine the effectiveness of cognitive behavioural therapy and determine moderators of treatment response. The study was pre-registered (PROSPERO 2020 CRD42020202776). Databases were systematically searched (PsycINFO, Medline, Embase, PubMed, ERIC) in December 2022 and screened according to the PRISMA 2020 statement. Inclusion: School aged participants (4–18 years) with a primary internalising disorder; psychotherapy delivered in a routine setting (e.g. outpatient clinic, school) by setting staff; compared psychotherapy to any control in a randomised controlled trial; reported pre-to-post or pre-to-follow-up comparisons on the primary disorder according to child, parent or independent evaluator report; and was published in English. Risk of bias was assessed using the ROB 2.0 Cochrane tool. Results were synthesised using random effects to pool estimates. Risk ratios were used to analyse dichotomous data and standardised mean differences (SMD) for continuous data. Forty-five studies were included (*N* = 4901 participants; *M* = 13 years; range 8–16; *SD* = 2.5). Nine used waitlist control, 17 treatment as usual, 4 placebo; 15 compared psychotherapy to active control. Psychotherapy was associated with small significant effects pre- to post-treatment compared to non-active controls for anxiety (SMD =  − 0.24 to 0.50) and depression (SMD =  − 0.19 to 0.34) with effects differing by informant. Psychotherapy led to small significant pre-to-post-benefits in youth internalising disorders in routine settings. Results are limited by reporter type and follow-up.

**Supplementary Information:**

The online version contains supplementary material available at 10.1007/s10567-023-00433-8.

## Introduction

Approximately 13% of young people worldwide are estimated to experience a mental health problem, with anxiety (6.5%) and depressive disorders (2.6%) being two of the most common (Polanczyk et al., 2015). Globally, mental health problems in youth cause the largest burden of disease (Gore et al., 2011) and their impact is projected to continue to increase (Baranne & Falissard, 2018). Along with neurodevelopmental and behavioural disorders, internalising disorders (anxiety and depression) are leading contributors to disease burden caused by mental health problems (Gore et al., 2011). A recent United Nations Children’s Fund report (2021) indicates that the annual loss in human capital due to mental health conditions in 0–19-year-olds is US$387.2 billion, with USD$340.2 billion reflecting disorders that include anxiety and depression. Effective and scalable treatments are a critical public health concern.

A large body of evidence has established that psychological therapies show efficacy in treating anxiety (Baker et al., 2021; Higa-McMillan et al., 2016; James et al., 2020; Reynolds et al., 2012; Weisz et al., 2017) and depression (Eckshtain et al., 2020; Weersing et al., 2017; Weisz et al., 2017) in children and adolescents. Cognitive behavioural therapy (CBT) produces increased anxiety remission compared to waitlist and attention control conditions, but the evidence is weaker when compared to treatment as usual (TAU; James et al., 2020; Weisz et al., 2017). Similarly, CBT is efficacious for the treatment of adolescent depression (Weersing et al., 2017), yet effects appear to be larger for interpersonal psychotherapy (Eckshtain et al., 2020). Therefore, CBT and interpersonal psychotherapy (for depression) are likely to be associated with improved symptoms for mood and anxiety disorders, and are ideal candidates for use in routine settings.

While research on the efficacy of treatments is critical, these studies have typically been conducted in research settings where expert clinicians deliver the treatments in ideal conditions to often restricted (i.e. highly selected) samples (Weisz et al., 2013). Following a systematic search of clinical trials on youth psychopathology, Weisz and colleagues (2005) found that fewer than 2% of all studies had involved youth referred for treatment (vs. recruited via advertisements) by practitioners (vs. research clinicians) in clinical practice settings (vs. research settings). Research in routine settings typically involves more heterogeneous samples and the expertise, training and supervision of therapists is more closely aligned with regular service delivery (Bauer et al., 2015) and these organisational, service delivery, and clientele-based differences have been shown to impact on effectiveness (Wuthrich et al., 2021). Understanding whether and which psychotherapies can effectively treat youth anxiety and depression in routine settings is a critical public health concern if treatment of these common and debilitating mental health conditions is to be implemented in mental health services.

Two recent systematic review and meta-analyses have started to address this gap. Bear et al. (2020) compared the effectiveness of treatment for anxiety and depression compared to TAU delivered in mental health services. The study found some evidence of effectiveness of treatment on the primary outcome at pre-post with a medium to large effect size (Hedges’ *g* =  − 0.74), and a large effect size (Hedges’ *g* =  − 0.87) at follow-up. However, effects were pooled across psychotherapy, pharmacotherapy, or combined treatments compared to TAU with differences in effect size found for informant type (i.e. pre-post effects differed for clinician assessors [− 1.3], youth [− 0.70] and parents [− 0.59]), and presenting problem type (i.e. at pre-post effects were largest for depression [− 0.89], then anxiety [− 0.66], and mixed [− 0.52]). However, it is unclear what the effectiveness is for psychological therapy when compared to a range of control conditions (e.g. TAU, waitlist, placebo, active), whether differences exist between setting types (such as schools, outpatient clinics, hospitals), disorder types and informant types in terms of outcomes on pre-post, follow-up, and remission status. It is also important to determine the effectiveness of psychological interventions when applied in routine settings by routine staff, not by research staff who are likely to differ from routine staff with respect to level of training, therapeutic preferences, and supervised practice (Wuthrich et al., 2021). Wergeland et al. (2021) examined the effectiveness of CBT specifically when applied by clinicians in routine settings in both controlled and uncontrolled trials and found a large effect size pre–post-treatment (Hedges’ *g* =  − 1.50); however, this was based on outcome measures pooled across clinician rating scales, self-report, and parent report, and pooled across controlled and uncontrolled trials. It is also not entirely clear if clinicians were employed by the services themselves or were externally employed research staff delivering the intervention within the setting; therefore, the methodological approaches limit an understanding of whether psychotherapy is more effective than other therapies and TAU in these settings.

Therefore, the aim of this systematic review and meta-analysis was to extend the literature to examine the effectiveness of psychotherapies delivered in routine care settings by setting staff to treat internalising disorders (anxiety and depressive disorders) in school-aged children (4–18 years) based on child, parent, and independent evaluator report on outcomes at pre-post, follow-up, and remission status. The second objective was to compare the effectiveness of traditional CBT (current best practice) delivered in routine care settings to the effectiveness of other psychological treatments delivered in routine care settings. The final objective was to examine potential moderators of these effects by type of control group and treatment setting.

## Method

### Protocol and Registration

A study protocol was registered and can be accessed at PROSPERO 2020 CRD42020202776. This review was conducted in accordance with the protocol and reported according to the Preferred Reporting Items for Systematic Reviews and Meta-Analyses (PRISMA) Statement 2020.

### Eligibility Criteria

For inclusion, studies had to be a randomised controlled trial (RCT) with school-aged children (4–18 years) with an internalising disorder as their primary diagnosis. Internalising disorders comprised DSM-IV and DSM-5 (American Psychiatric Association, 2000, 2013) anxiety and mood disorders including generalised anxiety disorder, social anxiety disorder, specific phobias, separation anxiety disorder, obsessive-compulsive disorder (OCD), posttraumatic stress disorder (PTSD), panic disorder, agoraphobia, not otherwise/other specified anxiety disorder, major depressive disorder, persistent depressive disorder and not otherwise/other specified depressive disorder, as determined by a diagnostic interview or clinical cut-off score on a validated measure. Studies involving participants with subclinical symptoms were excluded if they did not report outcomes separately for those with and without a clinical diagnosis. All interventions that incorporated a form of psychotherapy designed to reduce internalising symptoms were eligible if they were delivered in a routine setting (e.g. outpatient clinic, hospital, school) by setting staff (e.g. health-care clinician, school staff). If the intervention was delivered in a laboratory setting or by research personnel, or if there was insufficient information to determine the setting or personnel, the study was excluded. Psychotherapy could be compared to any control comparison i.e. another form of psychotherapy, waitlist control, no treatment, standard care, pharmacological or placebo (as indicated by the original study authors). The primary outcome of interest was a change in symptoms of anxiety or depression at post-treatment or follow-up as indicated by a change in diagnostic status (i.e. remission of primary disorder) and/or symptom severity. Only peer-reviewed articles written in English language were considered for inclusion.

### Selection Process

In March Week 2 of 2020, a systematic search for relevant studies was performed within the following databases: PsycINFO, Medline, Embase, PubMed and ERIC. The final search was conducted in December Week 3 of 2022. The search consisted of a combination of key terms, taking into account plurals (“adolescent/adolescence”) and synonyms (“community mental health services/community care”). See Online Appendix A for complete list of search terms for all databases. We used Covidence software as recommended by Cochrane for the screening process. Two independent authors (DZ, JC) screened titles and abstracts blindly against the inclusion criteria, and conflicts were resolved through discussion. Any studies that were not excluded by either reviewer were subject to a full-text review. Downloaded full texts were screened blindly and independently by the same reviewers, with a third independent reviewer (VW) acting as an arbiter if necessary.

### Data Extraction Variables

The following study characteristics were extracted into Microsoft Excel by one reviewer (DZ), and a second reviewer (JC) checked the study characteristics for accuracy against study reports.

- *Study* (1) author, (2) aims, (3) country of origin.
- *Participants* (4) number, (5) mean age, (6) age range, (7) % male, (8) diagnosis.
- *Interventions* (11) description, (12) setting, (13) who delivered intervention, (14) delivery format (group or individual), (15) mode (online or face to face), (16) length (weeks).

Treatment setting was classified as either school or non-school. When treatment was delivered across multiple settings, the prevailing setting, that is, the setting that saw the most treatments delivered, was used as the basis for classification.

*Comparators* (17) type (active, wait list, treatment as usual, placebo). Note as per the Cochrane handbook (McKenzie et al., 2022), we classified *inactive* treatment as comprising placebo, no treatment, waitlist and treatment as usual; and *active* treatment as variants of the same intervention, a drug and a different kind of psychological therapy. Where multiple treatment arms (e.g. psychotherapy versus medication versus control) were reported in the same study, only the relevant arms were analysed. When two or more relevant treatments were compared to controls (e.g. brief CBT versus standard CBT versus control), the data from the treatment that were anticipated to be superior were used. When two relevant treatments were compared without a control (e.g. prolonged exposure versus supportive counselling), data from the intervention that incorporated traditional CBT were considered the intervention, while data from the other type of psychotherapy were considered the comparison. When studies compared two variants of a CBT intervention, only they were not included in the meta-analysis.

*Outcomes* (18) outcome (anxiety or depression), (19) reporter (parent, child, independent evaluator [typically clinician rater blind to treatment allocation]), (20) measurement scale and (21) time points reported. Post-treatment was defined as the assessment administered closest to the end of treatment, and there were no restrictions on qualifying follow-up periods. When studies reported on the same outcome (e.g. anxiety) using multiple measurement scales, the most commonly used scale in the analysis was prioritised such that each study provided a single estimate per outcome. When studies reported on different outcomes (e.g. PTSD severity and depression) all outcomes were examined. When results were reported separately for mother and father report, priority was given the mothers’ report to maximise consistency. When clinician-administered interviews featured parent and child ratings, priority was given to the combined parent–child rating. When the combined parent–child rating was not reported, priority was given to the parent rating.

### Synthesis Methods

Statistical analysis was performed with RevMan software, Version 5.4 (Cochrane Collaboration, 2020). The generic inverse variance method was utilised, and a random effects model was used to pool the effect estimates for anxiety and depression outcomes. We analysed dichotomous data as risk ratios (RR). We analysed continuous data as standardised mean differences (SMD); that is, the mean difference between intervention and control groups from pre- to post-treatment, or pre-treatment to follow-up (where available) divided by the standard deviation (SD) of the outcome among participants (Cohen, 1988). We attempted to contact the original study authors for missing data. When the required outcome data were only available in figures, the data were extracted using ‘WebPlotDigitizer’ software (Rohatgi, 2021). When only standard errors (SE) were given, SDs were calculated by multiplying the SE by the square root of the sample size. When only 95% confidence intervals were supplied, SDs were calculated by dividing the length of the confidence interval by 3.92, and then multiplying by the square root of the sample size. One study (Parhiala et al., 2020) reported only the range at baseline, and in this instance the SDs were calculated by dividing the range of the data by four. Where only the pre-treatment or only the post-treatment SD was available, the missing SD was substituted by the other since it was reasonable to assume that the intervention did not alter the variability of the outcome measure. Where the SD of the mean difference was not given, calculation of the variance of within-group change scores from separate pre- and post-treatment standard deviations assumed a within-subject correlation of 0.5. The results of a sensitivity analysis around this correlation (using correlation values 0.3 and 0.7) were consistent with those of the main analysis. We considered trials in which participants with primary OCD or PTSD were recruited. The results of the sensitivity analysis hinted that the inclusion of PTSD – but not OCD – may have affected child-reported symptom outcomes (see Online Appendix B). We were interested in the differences between waitlist (WL) and treatment as usual (TAU) outcomes among studies that used inactive comparators. Three studies (Khanna & Kendall, 2010; Masia-Warner et al., 2016; Wright et al., 2020) used placebo controls, and we grouped them with the TAU controls because they were more similar with regard to their effect sizes. This decision was supported by a sensitivity analysis that excluded the three studies by: Khanna and Kendall (2010), Masia-Warner et al. (2016), and Wright et al. (2020).

When there were a minimum of 10 studies in the comparison, we generated funnel plots and ran regression tests (Egger et al., 1997) to assess for possible publication bias. When there is no bias the funnel plot will have an asymmetrical (inverted) funnel form and the p value of the intercept on Egger’s test will be equal to or above 0.1. Only when it was possible and meaningful to do so—i.e. when at least three studies contributed data to each subgroup—did we perform subgroup analysis by type of control group and treatment setting.

### Reporting Bias Assessment

All included studies were assessed with the revised “Risk of Bias” assessment tool Version 2.0 developed by the Cochrane Collaboration (Sterne et al., 2019), a scale measuring risk of bias on five different domains: (1) risk of bias arising from the randomisation process, (2) risk of bias due to deviations from the intended interventions, (3) risk of bias due to missing outcome data (when intent-to-treat analyses were conducted that included all randomised participants in the analyses we rated this as positive), (4) Risk of bias in measurement of the outcome (systematic differences between groups in how outcomes are assessed), and (5) risk of bias in selection of the reported result (deviations from the pre-specified analysis plan on the basis of the study results). Two independent raters (DZ, JC) responded to each risk of bias domain by indicating whether there was “low” or “high” risk of bias, or “some concerns”. Conflicts were resolved through discussion.

## Results

**Fig. 1:**
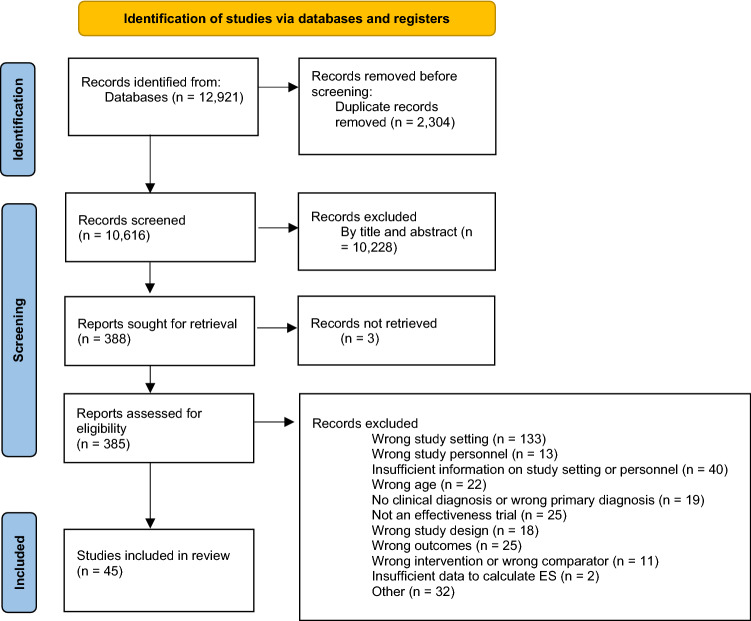
PRISMA flow diagram of literature search and study selection. *Note* From Page et al. (2021)

Figure 1 depicts the method for identifying, screening, and including studies. The database search identified 12,921 studies, and 2204 duplicates were found and removed. Of the 388 studies screened at full text, 343 did not meet the criteria. Two further studies appeared to meet all the inclusion criteria but were excluded because there were insufficient data to calculate effect sizes (Dorsey et al., 2020; Konanur et al., 2015). A total of 45 studies met criteria for inclusion in the review.

### Study Characteristics

The 45 RCTs included a total of 4901 participants (range = 20–403). Mean age of participants was 13 years (range = 8–16 years, *SD* = 2.5). Mean percentage of males was approximately 37.0% (range = 0%–67.3%). Twenty-seven studies were based in outpatient centres, 11 in schools, 3 in hospitals, and 4 in other settings (refugee camp, women’s shelter, rape crisis centre, residential treatment facility). Nine studies utilised a WL control, 17 studies utilised a TAU control, four studies compared against placebo therapy, and 15 studies compared psychotherapy to another active treatment of which 7 compared two different variants of CBT and 1 compared two active treatments where neither was classified as CBT. Interventions were delivered by a wide range of multidisciplinary clinicians (e.g. psychologists, social workers, mental health workers, primary care practitioners) employed in the setting it was delivered (e.g. teachers in schools, clinicians in Child & Adolescent Mental Health Services [CAMHS]), some used clinicians employed in the community who came into a different setting to deliver the interventions (e.g. CAMHS clinicians delivering the intervention in a hospital or school), two studies used an internet program with no therapist, and one study used trained lay-persons. TAU varied widely across studies with supportive counselling, counselling, self-help websites, CBT skills and access to pharmacotherapy included. Follow-up periods included 1 month (*n* = 1), 3 months (*n* = 2), 4 month (*n* = 1), 5 month (*n* = 1), 6 month (*n* = 12) and 12 month (*n* = 6). Characteristics of individual studies are summarised in Table 1.

**Table 1:** Characteristics of included studies

	Study	Country	Population		Intervention				Comparator Control	Outcome		
			*N*	Age (mean)	Therapy type	Target condition	Setting	Therapist		Measure	Scale	Report
1.	Auslander et al. (2017)	USA	34	14.6	CBT	PTSD	Outpatient	Service clinicians	TAU	PTSD, Depress	CPSS	Child
2.	Barrington et al. (2005)	AUS	54	10	CBT	Any anxiety	Hospital	CAMHS clinicians	TAU	Anx	RCMAS, SCAS-P, SCAS-C, BASC	Parent, Child
3.	Bodden et al. (2008)	NLD	147	12.4	CBT	Any anxiety	Outpatient	Service clinicians	Active	Anx	ADIS SCARED-P, SCARED-C, STAI-P STAI-C	IE, Parent, Child
4.	Catani et al. (2009)	LKA	31	11.9	CBT	PTSD	Other	Teachers	Active	PTSD	UPID	IE
5.	Chavira et al. (2014)	USA	48	9.6	CBT	Any anxiety	Outpatient	Community clinicians	Active	Anx	SCARED-P, SCARED-C, ADIS-C/P	Parent, Child, IE
6.	Cohen et al. (2004)	USA	229	10.76	CBT	PTSD	Outpatient	Service clinicians	Active	Anx Depress	STAI, CDI-C	Child
7.	Cohen et al. (2011)	USA	124	9.64	CBT	PTSD	Outpatient	Service clinicians	Active	PTSD, Anx, Depress	K-SADS, RI, SCARED-C, CDI-C	IE, Child
8.	Creswell et al. (2017)	GBR	136	9.2	CBT	Any anxiety	Outpatient	Service clinicians	Active	Anx	SCAS-P, SCAS-C, KFQ, ADIS-C/P	Parent, Child, IE
9.	Dawson et al. (2018)	IDN	64	10.7	CBT	PTSD	School	CAMHS staff	Active	PTSD, Depress	UPID-P, UPID-C, CDI-C	Parent, Child
10.	Foa et al. (2013)	USA	61	15.3	PE	PTSD	Other	Service clinicians	Active	PTSD, Depress	CPSS, CPSS-C, CDI-C	IE, Child
11.	Ginsburg et al. (2012)	USA	32	10.3	CBT	Any anxiety	School	School clinicians	TAU	Anx	ADIS, SCARED-P, SCARED-C	IE, Parent, Child
12.	Ginsburg et al. (2020)	USA	216	10.87	CBT	Any anxiety	School	School clinicians	TAU	Anx	SCARED-P, SCARED-C	Parent, Child
13.	Goldbeck et al. (2016)	DEU	159	13.03	Tf-CBT	PTSD	Outpatient	Service clinicians	WL	PTSD, Depress, Anx	CAPS-CA, UPID-P, UPID-C, CPTCI, CDI-C	IE, Parent, Child
14.	Gordon et al. (2008)	XXK	82	16.3	Mindfulness	PTSD	School	Teachers	WL			
15.	Hateli (2022)	IRN	20	7.85	PT	Any anxiety	Outpatient	Service clinicians	WL	Anx	SCAS-P	Parent
16.	Haugland et al. (2020)	NOR	313	14	CBT	Any anxiety	School	Community clinicians	Active	Anx, Depress	SCAS-P, SCAS-C, SMFQ-C, SMFQ-P	Parent, Child
17.	Israel and Diamond (2013)	NOR	20	15.6	ABFT	Mood	Hospital	Service clinicians	TAU	Depress	HDI, BDI	IE, Child
18.	Jensen et al. (2014)	NOR	156	15.1	CBT	PTSD	Outpatient	Service clinicians	TAU	PTSD	CAPS-CA	IE
19.	Jeppesen et al. (2021)	DNK	396	10.3	CBT	Any anxiety, mood or behaviour	Other	Community clinicians	TAU	Anx, Depress	SCAS-P, MFQ-P	Parent
20.	Kerfoot et al. (2004)	GBR	42	13.9	CBT	Mood	Outpatient	Community clinicians	TAU	Depress	MFQ-C	Child
21.	Khanna and Kendall (2010)	USA	49	10.1	CBT	Any anxiety	Outpatient	Community clinicians	Plac	Anx, Depress	ADIS-C/P, MASC, CDI-C	IE, Child
22.	Kitchen et al. (2021)	UK	22	16	BA	Mood	Outpatient	CAMHS staff	TAU	Depress	MFQ-P MFQ-C	Parent, Child
23.	Lau et al. (2010)	CHN	45	8.4	CBT	Any anxiety	Outpatient	Service clinicians	WL	Anx	SCAS-C, SCAS-C	Parent, Child
24.	Lorentzen et al. (2020)	NOR	163	15.72	CBT	Anxiety or mood	Outpatient	Service clinicians	WL	Anx, Depress	MASC, BDI	Child,
25.	Masia-Warner et al. (2016)	USA	73	15.42	CBT	Social Anxiety Disorder	School	School clinicians	Plac	Anx	ADIS-C/P, SPAI-P, SPAI-C, LSAS-CA	IE, Parent, Child
26.	Melvin et al. (2006)	AUS	64	15.3	CBT	Mood	Outpatient	Service clinicians	Active	Anx, Depress	RADS, RCMAS	Child
27.	Mufson et al. (2004)	USA	52	15.1	IP	Mood	School	School clinicians	TAU	Depress	HAMD, BDI	IE, Child
28.	O’Callaghan et al. (2013)	COG	55	16.02	CBT	PTSD	Other	Community clinicians	WL	PTSD	UPID-C	Child
29.	Parhiala et al. (2020)	FIN	50	14.5	IP	Mood	School	School clinicians	TAU	Depress	BDI, ADRS	Child, IE
30.	Reynolds et al. (2013)	GBR	42	14.5	CBT	OCD	Outpatient	Service clinicians	Active	OCD, Anx, Depress	CYBOCS, BAI, SMFQ	IE, Child,
31.	Riemann et al. (2013)	USA	63	15.57	CBT + AM	Any anxiety	Outpatient	Service clinicians	Plac	Anx, Depress, OCD	SCARED-C, BDI, CYBOCS-C	Child
32.	Shechner et al. (2014)	ISR	43	11.5	CBT + ABMT	Any anxiety	Outpatient	Service clinicians	Active	Anx	ADIS-C/P	IE
33.	Shirk et al. (2014)	USA	112	15.48	CBT	Mood	Outpatient	Service clinicians	TAU	Depress	BDI	Child
34.	Smith et al. (2015)	GBR	48	NR	CBT	Mood	School	Nil (internet delivered)	Active	Anx, Depress	MFQ-C, MFQ-P, SCARED-C	Child, Parent
35.	Southam-Gerow et al. (2010)	USA	100	10.9	CBT	Any anxiety	Outpatient	Service clinicians	TAU	Anx, Depress	STAI-P STAI-C DISC-Anx -P/C/P + C DISC-Dep-P/C/P + C	Parent, Child, IE
36.	Storch et al. (2015)	USA	403	9.8	CBT	Any anxiety	Outpatient	Service clinicians	TAU	Anx, Depress	PARS, ADIS-C/P, CDI-C, MASC	IE, Child
37.	Tol et al. (2008)	IDN	72	9.94	Other	PTSD	School	Trained layperson	WL	PTSD, Depress, Anx	CPSS-C, DSS, SCARED-C	Child
38.	Trowell et al. (2007)	GBR, GRC, FIN	72	11.71	IPDS	Mood	Hospital, Outpatient	Service clinicians	Active	Depress	CDI-C, MFQ	Child
39.	Turner et al. (2014)	AUS	165	14.3	CBT	OCD	Outpatient	Service clinicians	Active	OCD, Depress	CYBOCS, ChOCI-R-P, ChOCI-R–C, BDI	IE, Parent, Child,
40.	Villabø et al. (2018)	NOR	60	10.5	CBT	Any anxiety	Outpatient	Service clinicians	WL	Anx	MASC-P, MASC-C	Parent, Child
41.	Waraan et al. (2021)	NOR	138	14.9	ABFT	Mood	Outpatient	Service clinicians	TAU	Depress	HAMD, BDI	IE, Child
42.	Weisz et al. (2009)	USA	57	11.77	CBT	Mood	Outpatient	Service clinicians	TAU	Depress	CDI-P, CDI -C DISC-Dep-C, DISC-Dep-P,	Parent, Child
43.	Wergeland et al. (2021)	NOR	182	11.5	CBT	Any anxiety	Outpatient	Service clinicians	WL	Anx, Depress	ADIS-C/P, SCAS-P, SCAS-C, SMFQ-P, SMFQ-C	IE, Parent, Child
44.	Wright et al. (2020)	GBR	139	15	CBT	Mood	School, Outpatient	Nil (internet delivered)	Plac	Anx, Depress	BDI, SCAS-C	Child
45.	Wright et al. (2022)	UK	268	11.8	OST	Phobia	Outpatient	Service clinicians	TAU	Anx	ADIS-C/P	Clinician

*NR* not reported, *AUS* Australia, *DNK* Denmark, *USA* United States of America, *GBR* United Kingdom, *NLD* Netherlands, *LKA* Sri Lanka, *NOR* Norway, *IDN* Indonesia, *DEU* Germany, *XXK* Kosovo, *CHN* China, *IRN* Iran, *COG* the Democratic Republic of the Congo, *FIN* Finland, *ZAF* South Africa, *ISR* Israel, *GRC* Greece, *CAMHS* Child & Adolescent Mental Health Service, *Anx* Anxiety, *Depress* Depression, *CBT* cognitive behavioural therapy, *ABMT* Attention Bias Modification Treatment, *PE* Prolonged Exposure, *tf-CBT* trauma-focused CBT, *AM* attention modification, *ABFT* Attention based family therapy, *IP* interpersonal psychotherapy, *IPDS* individual psycho-dynamic psychotherapy, *PT* play therapy, *BA* behaviour activation, *OST* One-session treatment, *Ind* individual, *WL* waitlist, *TAU* treatment as usual, *Plac* placebo, *CPSS* Child PTSD Symptom Scale, *RCMAS* Revised Children's Manifest Anxiety Scale, *SCAS-C/P* Spence Children’s Anxiety Scale-parent/child, *BASC* The Behaviour Assessment System for Children, *SCARED-P/C* Screen for Child Anxiety Related Emotional Disorders, *STAI-P/C* State Trait Anxiety Inventory-parent/child, *UPID-C/P* UCLA PTSD Index-parent/child, *ADIS-C/P* Anxiety Disorders Interview Schedule for Children-parent/child, *CDI-C/P* Children’s Depression Inventory-parent/child, *K-SADS* Kiddie Schedule for Affective Disorders and Schizophrenia, *RI* University of California at Los Angeles PTSD Reaction Index, *KFQ* Koala Fear Questionnaire, *CAPS-CA* Clinician-Administered PTSD Scale for Children and Adolescents, *CPTCI* Child Posttraumatic Cognitions Inventory, *MFQ-P/C* Mood and Feelings Questionnaire-parent/child, *SMFQ-C/P* Short Mood and Feelings Questionnaire, *HDI* Hamilton Depression Inventory, *BDI* Beck Depression Inventory, *MASC-P/C* Multidimensional Anxiety Scale for Children-parent/child, *LSAS-CA* Liebowitz Social Anxiety Scale for Children and Adolescents, *RADS* Reynolds Adolescent Depression Scale, *HAMD* Hamilton Depression Rating Scale, *ADRS* Adolescent Depression Rating Scale, *CYBOCS-C* Children’s Yale-Brown Obsessive Scale-child, *BAI-C* Beck Anxiety Inventory-child, *DISC-Anx/Dep/C/P/C*+*P* Diagnostic Interview Schedule for Children-Anxiety/Depression-parent/child/combined report, *PARS* Pediatric Anxiety Rating Scale, *ChOCI-R* Children’s Obsessional Compulsive Inventory-Revised, *DSS* Depression Self-Rating Scale, *IE* independent evaluator

### Risk of Bias in Studies

**Fig. 2:**
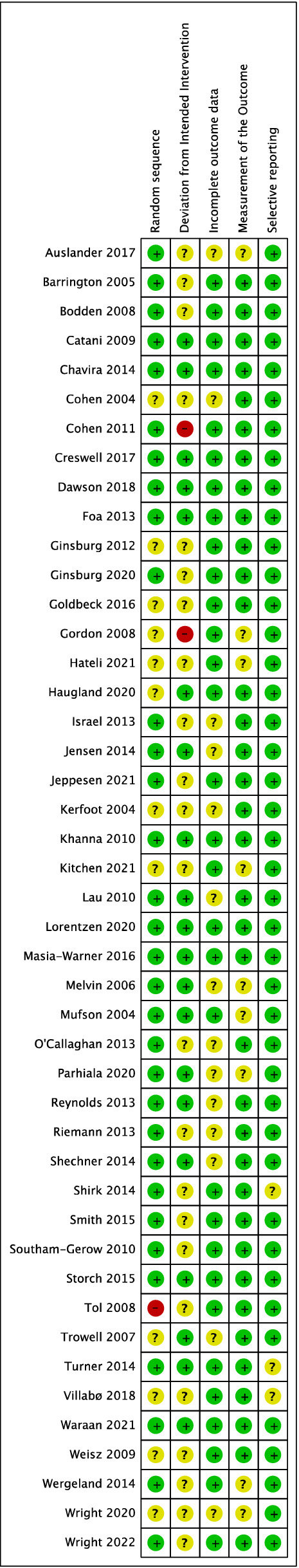
Risk of bias summary: review authors’ judgements about each risk of bias item for each included study

The risk of bias judgements for each study and for each category of bias are presented in Fig. 2.

### Outcome 1: Change in Anxiety Symptoms

#### Psychotherapy vs. Non-active Controls Pre–post-Treatment

**Fig. 3:**
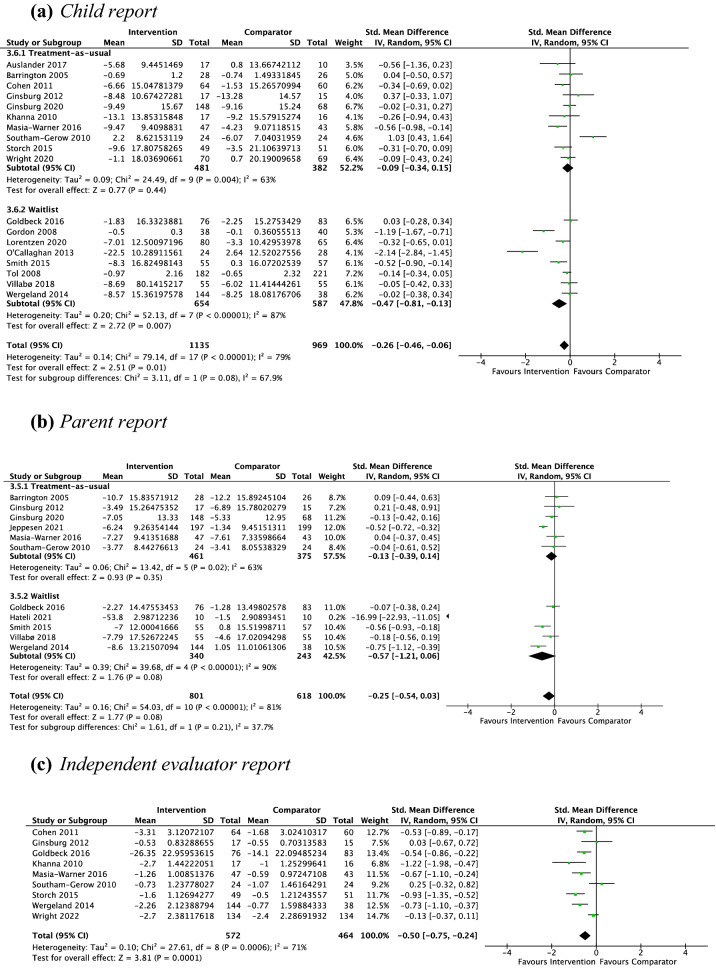
Outcome 1: psychotherapy vs. non-active controls: change in anxiety symptoms at post-treatment

*Child Report* Eighteen studies, including 2104 participants revealed a significant small effect of psychotherapy on child-reported anxiety symptoms of − 0.26 (95% CI [− 0.06, − 0.46], *Z* = 2.51, *p* = 0.01), with substantial heterogeneity given by *I*^2^ of 79% (see Fig. 3a). The test for subgroup differences in outcomes by type of control group indicated no significant subgroup effect (*p* = 0.08). The test for subgroup differences in outcomes according to treatment setting indicated no statistically significant subgroup effect (*p* = 0.70, Online Appendix C1).

*Parent Report* Eleven studies, including 1419 participants revealed a nonsignificant small effect of psychotherapy on parent-reported anxiety symptoms of − 0.25 (95% CI [− 0.03, − 0.54], *Z* = 1.77, *p* = 0.08), with substantial heterogeneity given by *I*^2^ of 81% (see Fig. 3b). The test for subgroup differences in outcomes by type of control group indicated no significant subgroup effect (*p* = 0.21). When Hateli (2022) was removed as an outlier, the effect size changed to − 0.24 (*p* = 0.01). The test for subgroup differences in outcomes by treatment setting indicated no significant subgroup effect (*p* = 0.48; Online Appendix C2).

*Independent evaluator report* Nine studies, including 1036 participants revealed a significant medium effect of psychotherapy on independent evaluator-reported anxiety symptoms of − 0.50 (95% CI [− 0.24, − 0.75], *Z* = 3.81, *p* < 0.001), with substantial heterogeneity given by *I*^2^ of 71% (see Fig. 3c). No subgroup analysis was undertaken due to the small number of contributing studies once the data were separated by control group (TAU = 7, WL = 2) and treatment setting (school = 2, non-school = 7).

#### Psychotherapy vs. Non-active Controls Pre-treatment to Follow-Up

**Fig. 4:**
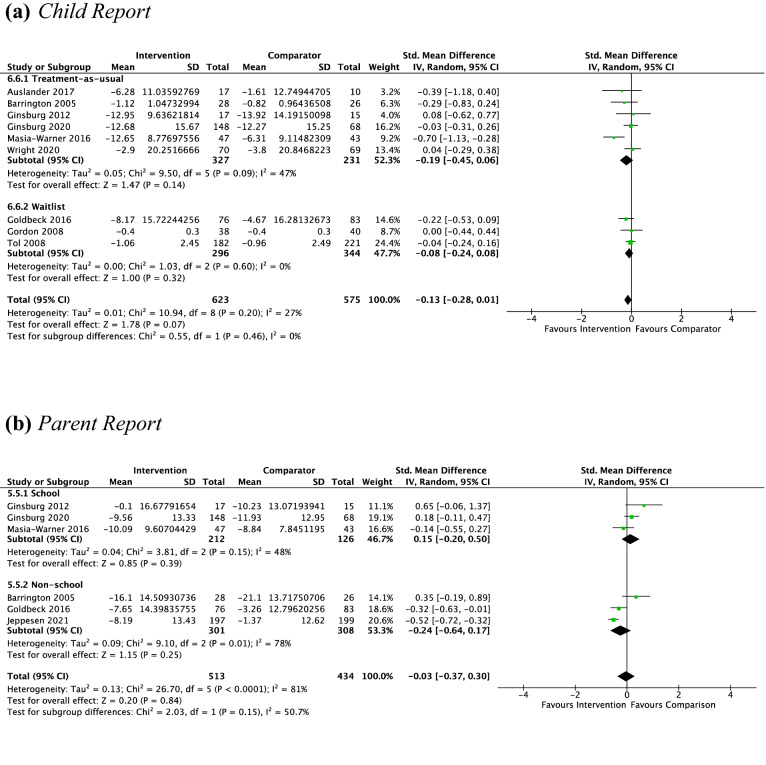
Outcome 1: psychotherapy vs. non-active controls: change in anxiety symptoms at follow-up

*Child Report* Nine studies, including 1198 participants revealed a nonsignificant small effect of psychotherapy on child-reported anxiety symptoms from pre-treatment to follow-up of − 0.13 (95% CI [0.01, − 0.28], *Z* = 1.78, *p* = 0.07), with moderate heterogeneity given by *I*^2^ of 27% (see Fig. 4a). The test for subgroup differences in outcomes according to control group indicated no significant subgroup effect (*p* = 0.46). No subgroup analysis was undertaken for the comparison of treatment settings due to the small number of contributing studies once the data were separated by treatment setting (school = 7, non-school = 2).

*Parent Report* Six studies, including 947 participants revealed a nonsignificant small effect of psychotherapy on parent-reported anxiety symptoms from pre-treatment to follow-up of − 0.03 (95% CI [0.30, − 0.37], *Z* = 0.20, *p* = 0.84), with considerable heterogeneity given by *I*^2^ of 81% (see Fig. 4b). The test for subgroup differences in outcomes according to treatment setting indicated no significant subgroup effect (*p* = 0.15). No subgroup analysis was undertaken for the comparison of control groups due to the small number of contributing studies once the data were separated by control group (TAU = 5, WL = 1).

*Independent evaluator report* Due to the low number of studies providing data to this outcome (*n* = 2), no analysis was performed.

#### CBT vs. Other Type of Psychotherapy Pre–post-treatment

**Fig. 5:**
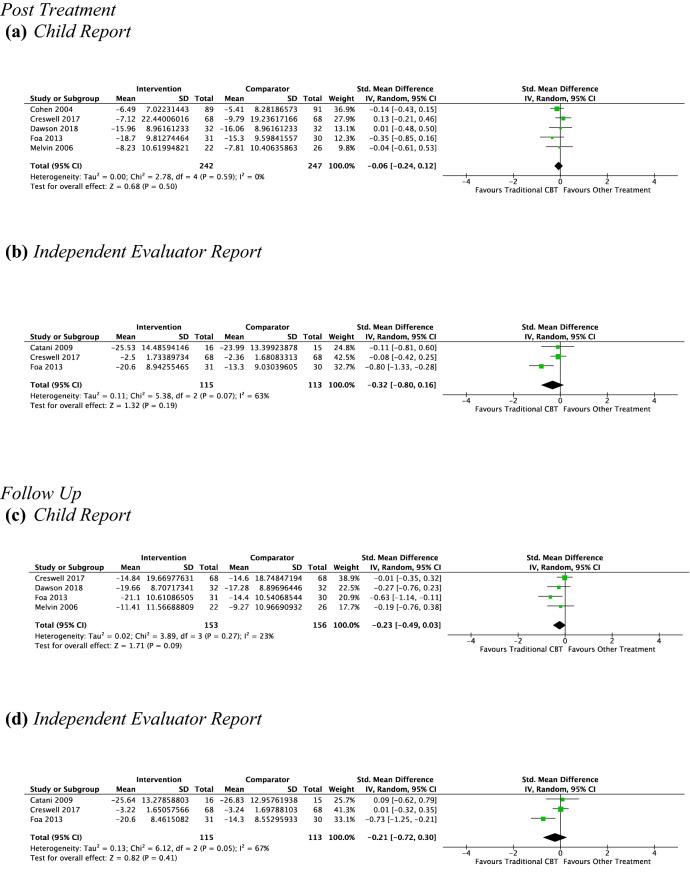
Outcome 1: CBT vs other type of psychotherapy: change in anxiety symptoms at post-treatment and at follow-up

*Child Report* Five studies, including 489 participants revealed a nonsignificant small effect of CBT on child-reported anxiety symptoms of − 0.06 (95% CI [0.12, − 0.24], *Z* = 0.68, *p* = 0.5), with unimportant heterogeneity given by *I*^2^ of 0% (see Fig. 5a). No subgroup analysis was undertaken due to the small number of contributing studies once the data were separated by treatment setting (school = 1, non-school = 4).

*Parent Report* Due to the low number of studies providing data to this outcome (*n* = 2), no analysis was performed.

*Independent evaluator report* Three studies, including 228 participants revealed a nonsignificant small effect of CBT on independent evaluator-reported anxiety symptoms of − 0.32 (95% CI [0.16, − 0.80], *Z* = 1.32, *p* = 0.19), with substantial heterogeneity given by *I*^2^ of 63%. See Fig. 5b. No subgroup analysis was undertaken due to the small number of studies contributing data to this outcome.

#### CBT vs. Other Type of Psychotherapy at Follow-Up

*Child Report* Four studies, including 309 participants revealed a nonsignificant small effect of CBT on child-reported anxiety symptoms at follow-up of − 0.23 (95% CI [0.03, − 0.49]; *Z* = 1.71, *p* = 0.09), with unimportant heterogeneity given by *I*^2^ of 23% (see Fig. 5c). No subgroup analysis was undertaken due to the small number of studies contributing data to this outcome.

*Parent report* Due to the low number of studies providing data to this outcome (*n* = 2), no analysis was performed.

*Independent evaluator report* Three studies, including 228 participants revealed a nonsignificant small effect of CBT on independent evaluator-reported anxiety symptoms at follow-up of − 0.21 (95% CI [0.30, − 0.72], *Z* = 0.82, *p* = 0.41) with substantial heterogeneity given by *I*^2^ of 67%. See Fig. 5d. No subgroup analysis was undertaken due to the small number of studies contributing data to this outcome.

### Outcome 2: Remission of Primary Anxiety Disorder

#### Psychotherapy vs. Non-active Controls at Post-treatment

*Child report* Due to the low number of studies providing data to this outcome (*n* = 1), no analysis was performed.

*Parent report* Due to the low number of studies providing data to this outcome (*n* = 1), no analysis was performed.

**Fig. 6:**
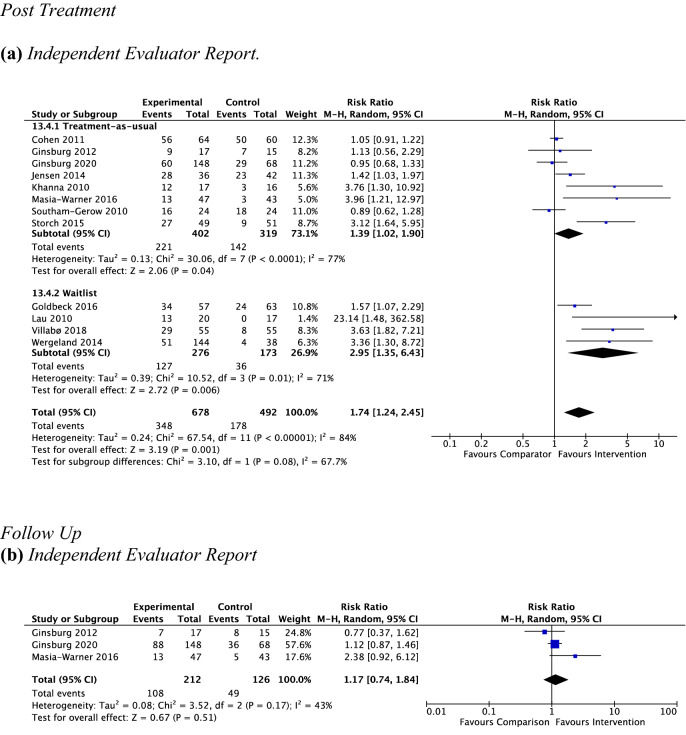
Outcome 2: psychotherapy vs. non-active controls: remission of primary anxiety disorder at post-treatment and follow-up

*Independent evaluator report* Twelve studies, including 1170 participants revealed a significant response rate for remission of primary anxiety diagnosis of 51.3% (348 out of 678) for psychotherapy versus 36.2% (178 out of 492) for controls (RR 1.74, 95% CI [1.24, 2.45], *Z* = 3.19, *p* = 0.001), with substantial heterogeneity given by *I*^2^ of 84%. The test for subgroup differences in outcomes according to type of control group indicated no significant subgroup effect (*p* = 0.08; see Fig. 6a). The test for subgroup differences in outcomes according to type of treatment setting indicated no significant subgroup effect (*p* = 0.34; Online Appendix C3).

#### Psychotherapy vs. Non-active Controls at Follow-Up

*Child report* Due to the low number of studies providing data to this outcome (*n* = 1), no analysis was performed.

*Parent report* Due to the low number of studies providing data to this outcome (*n* = 1), no analysis was performed.

*Independent evaluator report* Three studies, including 338 participants revealed a nonsignificant response rate for remission of primary anxiety diagnosis at follow-up of 50.9% (108 out of 212) for psychotherapy versus 38.9% (49 out of 126) for controls (RR 1.17, 95% CI [0.74, 1.84], *Z* = 0.67, *p* = 0.51), with moderate heterogeneity given by *I*^2^ of 43%. See Fig. 6b. No subgroup analysis was undertaken due to the low number of studies contributing data to this outcome.

#### CBT vs. Other Type of Psychotherapy at Post-treatment

*Child report* No analysis was performed as no studies provided data to this outcome.

*Parent report* No analysis was performed as no studies provided data to this outcome.

**Fig. 7:**
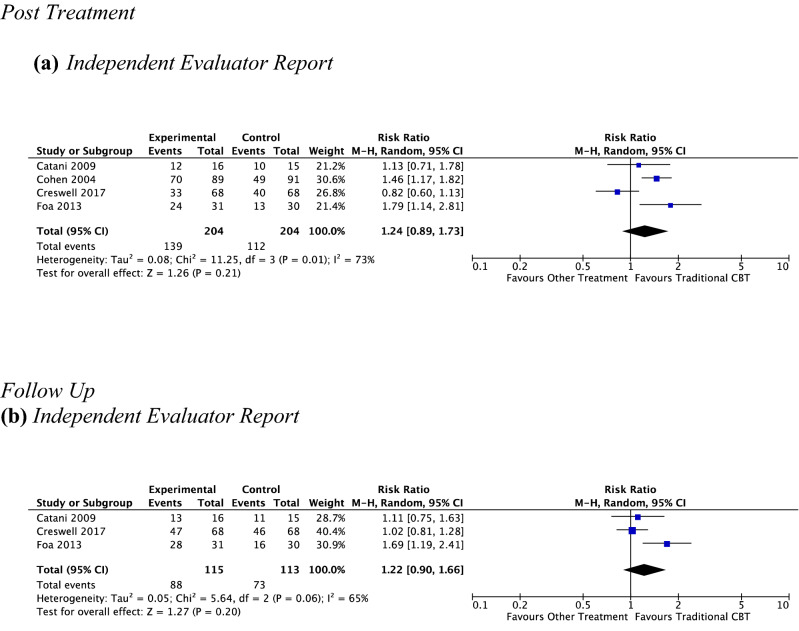
Outcome 2: CBT vs other type of psychotherapy: remission of primary anxiety disorder at post-treatment and at follow-up

*Independent evaluator report* Four studies, including 408 participants revealed a nonsignificant response rate for remission of primary anxiety diagnosis of 68.1% (139 out of 204) for CBT versus 54.9% (112 out of 204) for other treatment (RR 1.24, 95% CI [0.89, 1.73], *Z* = 1.26, *p* = 0.21), with substantial heterogeneity given by *I*^2^ of 73% (see Fig. 7a). No subgroup analysis was undertaken due to the small number of studies contributing data to this outcome.

#### CBT vs. Other Type of Psychotherapy at Follow-Up

*Child report* No analysis was performed as no studies provided data to this outcome.

*Parent report* No analysis was performed as no studies provided data to this outcome.

*Independent Evaluator Report* Three studies, including 228 participants, revealed a nonsignificant response rate for remission of primary anxiety diagnosis of 76.5% (88 out of 115) for CBT versus 64.6% (73 out of 113) for other treatment (RR 1.22, 95% CI [0.90, 1.66], *Z* = 1.27, *p* = 0.20), with substantial heterogeneity given by *I*^2^ of 65%. See Fig. 7b. No subgroup analysis was undertaken due to the small number of studies contributing data to this outcome.

### Outcome 3: Change in Depressive Symptoms

#### Psychotherapy vs. Non-active Controls Pre–post-treatment

**Fig. 8:**
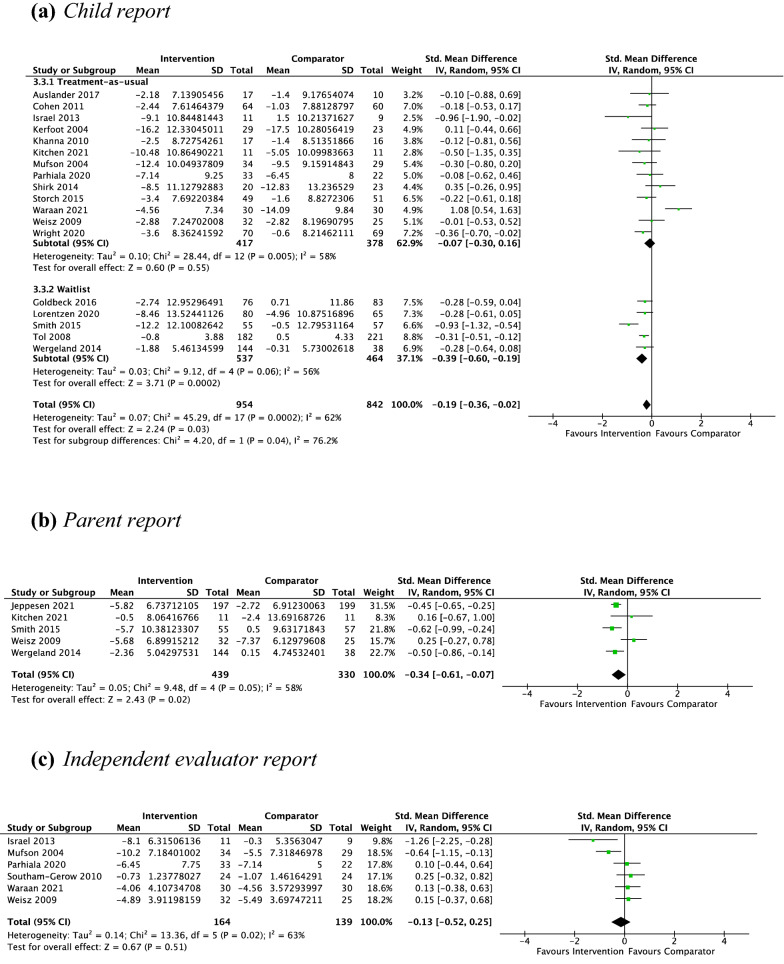
Outcome 3: psychotherapy vs. non-active controls: change in depressive symptoms at post-treatment

*Child report* Eighteen studies, including 1796 participants revealed a significant small effect of psychotherapy on child-reported depressive symptoms of − 0.19 (− 0.02, − 0.36; Z = 2.24, *p* = 0.03), with substantial heterogeneity given by *I*^2^ of 62%. The test for subgroup differences in outcomes according to type of control group indicated a significant subgroup effect (*p* = 0.04; see Fig. 8a). The treatment effect favoured psychotherapy over the waitlist control group (*d* =  − 0.39, *p* =  < 0.001) but not the treatment-as-usual group (*d* =  − 0.07, *p* = 0.55). The test for subgroup differences in outcomes according to treatment setting indicated no significant subgroup effect (*p* = 0.05, Online Appendix C4).

*Parent report* Five studies, including 769 participants revealed a significant small effect of psychotherapy on parent-reported depressive symptoms of − 0.34 (95% CI [− 0.07, − 0.61], *Z* = 2.43, *p* = 0.02), with substantial heterogeneity given by *I*^2^ of 58% (see Fig. 8b). No subgroup analysis was undertaken due to the small number of studies contributing data to this outcome.

*Independent evaluator report* Six studies, including 303 participants revealed a nonsignificant small effect of psychotherapy on independent evaluator-reported depressive symptoms of − 0.13 (95% CI [0.25, − 0.52], *Z* = 0.67, *p* = 0.51), with substantial heterogeneity given by *I*^2^ of 63% (see Fig. 8c). No subgroup analysis was undertaken due to the small number of contributing studies once the data were separated by control group (WL = 0, TAU = 6) and treatment setting (school = 2, non-school = 4). Note: all non-active controls were TAU.

#### Psychotherapy vs. Non-active Controls Pre-treatment to Follow-Up

**Fig. 9:**
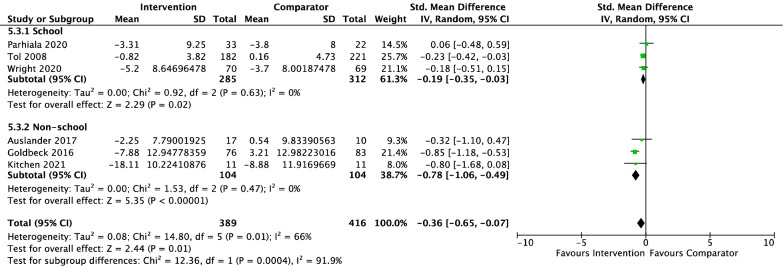
Outcome 3: psychotherapy vs. non-active controls: change in depressive symptoms at follow-up. Child report

*Child report* Six studies, including 805 participants revealed a significant small effect of psychotherapy on child-reported depressive symptoms from pre-treatment to follow-up of − 0.36 (95% CI [− 0.07, − 0.65], *Z* = 2.44, *p* = 0.01), with substantial heterogeneity given by *I*^2^ of 66%. The test for subgroup differences in outcomes according to treatment setting indicated a significant subgroup effect (*p* < 0.001; see Fig. 9). The treatment effect favoured psychotherapy for both school and non-school settings, although the treatment effect was greater for non-school settings (*d* =  − 0.78) than school settings (*d* =  − 0.19). No subgroup analysis was undertaken for the comparison of control groups due to the small number of contributing studies once the data were separated by control group (TAU = 4, WL = 2).

*Parent report* Due to the low number of studies providing data to this outcome (*n* = 2), no analysis was performed.

*Independent evaluator report* Due to the low number of studies providing data to this outcome (*n* = 1), no analysis was performed.

#### CBT vs. Other Type of Psychotherapy at Post-treatment

**Fig. 10:**
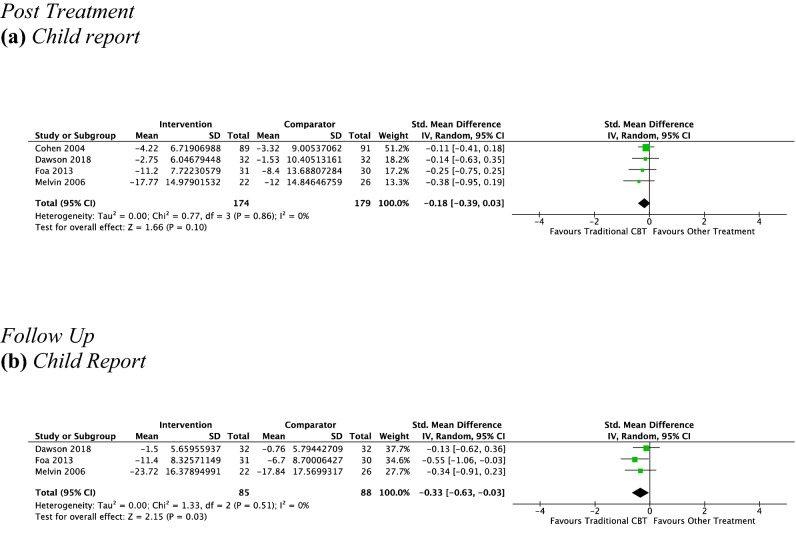
Outcome 3: CBT vs. other type of psychotherapy: change in depressive symptoms at post-treatment and follow-up

*Child report* Four studies, including 353 participants, revealed a nonsignificant small effect of traditional CBT on child-reported depressive symptoms of − 0.18 (95% CI [0.03, − 0.39], *Z* = 1.66, *p* = 0.1), with unimportant heterogeneity given by *I*^2^ of 0% (see Fig. 10a). No subgroup analysis was undertaken due to the small number of studies contributing data to this outcome.

*Parent report* No analysis was performed as no studies provided data to this outcome.

*Independent Evaluator Report* No analysis was performed as no studies provided data to this outcome.

#### CBT vs. Other Type of Psychotherapy at Follow-Up

*Child report* Three studies, including 173 participants revealed a significant small effect of psychotherapy on child-reported depressive symptoms from pre-treatment to follow-up of − 0.33 (95% CI [− 0.03, − 0.63], *Z* = 2.15, *p* = 0.03), with unimportant heterogeneity given by *I*^2^ of 0%. See Fig. 10b. No subgroup analysis was undertaken due to the small number of studies contributing data to this outcome.

*Parent Report* No analysis was performed as no studies provided data to this outcome.

*Independent Evaluator Report* No analysis was performed as no studies provided data to this outcome.

### Outcome 4: Remission of Primary Depressive Disorder

#### Psychotherapy vs. Non-active Controls at Post-treatment

**Fig. 11:**
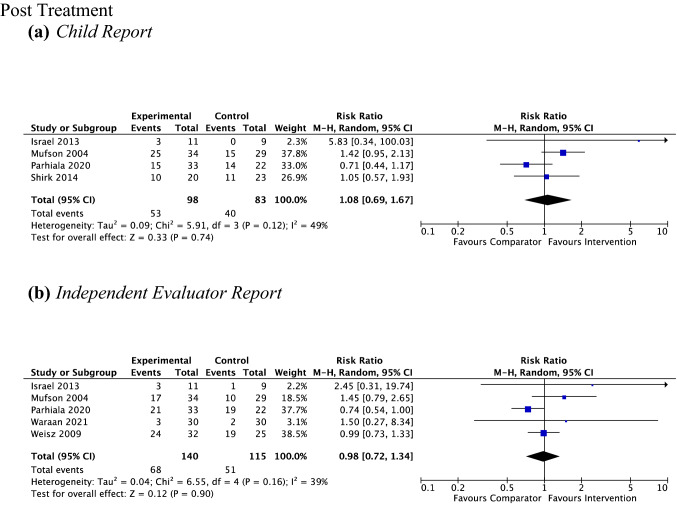
Outcome 4: psychotherapy vs non-active control: remission of primary depressive disorder at post-treatment and follow-up

*Child Report* Four studies, including 181 participants revealed a nonsignificant response rate for remission of primary depressive diagnosis of 54.1% (53 out of 98) for psychotherapy versus 48.2% (40 out of 83) for controls (RR 1.08, 95% CI [0.69, 1.67], *Z* = 0.33, *p* = 0.74), with moderate heterogeneity given by *I*^2^ of 49% (see Fig. 11a). No subgroup analysis was undertaken due to the small number of studies contributing data to this outcome.

*Parent report* No analysis was performed as no studies provided data to this outcome.

*Independent evaluator report* Five studies, including 255 participants revealed a nonsignificant response rate for remission of primary depressive diagnosis of 48.6% (68 out of 140) for psychotherapy versus 44.3% (51 out of 115) for controls (RR 0.98, 95% CI [0.72, 1.34], *Z* = 0.12, *p* = 0.90), with moderate heterogeneity given by *I*^2^ of 39% (see Fig. 11b). No subgroup analysis was undertaken due to the small number of studies contributing data to this outcome.

#### Psychotherapy vs. Non-active Controls at Follow-Up

*Child report* No analysis was performed as no studies provided data to this outcome.

*Parent report* No analysis was performed as no studies provided data to this outcome.

*Independent evaluator report* No analysis was performed as no studies provided data to this outcome.

#### CBT vs. Other Type of Psychotherapy at Post-treatment

*Child report* No analysis was performed as no studies provided data to this outcome.

*Parent report* No analysis was performed as no studies provided data to this outcome.

*Independent evaluator report* No analysis was performed as no studies provided data to this outcome.

#### CBT vs. Other Type of Psychotherapy at Follow-Up

*Child report* No analysis was performed as no studies provided data to this outcome.

*Parent report* No analysis was performed as no studies provided data to this outcome.

*Independent evaluator report* No analysis was performed as no studies provided data to this outcome.

### Publication Bias

Funnel plots (see Appendices D–G) were used to detect asymmetry, potentially evidencing publication bias. The plot for child-reported anxiety symptoms showed no indication of asymmetry, which was supported by the nonsignificant result of Egger’s test (*Z* =  *− *0.90, *p* = 0.37). There was some hint of asymmetry in the plots for parent-reported anxiety symptoms and child-reported depressive symptoms; however, the nonsignificant Egger’s test results (*Z* =  − 1.41, *p* = 0.16; *Z* = 0.85, *p* = 0.40, respectively) did not support this interpretation. There was clear indication of asymmetry in the plot for clinician-reported anxiety remission, which was supported by a significant Egger’s test result (*Z* = 2.91, *p* =  < 0.01).

## Discussion

Populations who are recruited within typical randomised controlled trials for the treatment of paediatric anxiety and depression are usually conducted in research settings, and their samples are commonly more educated, wealthier, and racially homogenous than the general population and especially relative to populations seeking treatment from community settings (Ehrenreich-May et al., 2011; James et al., 2020). Professionals that deliver interventions in routine settings are also likely to differ in qualifications, therapeutic preferences, and frequency of peer supervision received (Wuthrich et al., 2021). Therefore, it cannot be assumed that outcomes from typical research trials will generalise to delivery within broader community settings. The current study aimed to evaluate this question of effectiveness rather than efficacy using strict criteria to ensure the clinicians worked in the community settings and that interventions were not delivered by external research staff. We examined effects against non-active controls (which as defined by the Cochrane Handbook includes placebo, no treatment, waitlist and treatment as usual) and active controls (that included variants of therapies and pharmacotherapy).

In this study, the majority of included studies used child-reported outcomes from pre-to-post-treatment to evaluate outcomes in community settings, with inconsistent inclusion of parent and independent evaluator reporting of outcomes. Fewer studies examined changes from pre to follow-up periods (with follow-up periods varying from 1 to 12 months) limiting conclusions that can be drawn as to maintenance of effects. Fewer studies examined changes in remission status over time. This reflects differences in the routine assessment approaches typically used in community settings.

Overall, the results indicated that current psychotherapies for anxiety and depression in youth are partially effective when delivered in routine settings compared to non-active controls, with some differences in effect depending on problem type (depression vs. anxiety), the informant, and whether post or follow-up treatment period was examined (as previously found by Bear et al., 2020; De Los Reyes & Kazdin, 2005; Eckshtain et al., 2020; Weisz et al., 2017). Psychotherapy was associated with significant small to medium effects for reduction in anxiety symptoms according to child report (ES =  − 0.26), parent report (ES =  − 0.25) and independent evaluator report (ES =  − 0.50) from pre-to-post-treatment over non-active controls. However, effects were nonsignificant for child and parent report from pre to follow-up, and were unable to be tested for independent evaluator reports. Remission status for anxiety symptoms could only be examined according to independent evaluator report and findings indicated significant small effects for improved remission of anxiety disorders from pre- to post-treatment for psychotherapy over non-active controls, but nonsignificant effects from pre to follow-up matching the pattern of findings for anxiety symptoms.

Similarly, when symptoms of depression were the target, psychotherapy was associated with small significant effects over non-active interventions from pre- to post-treatment according to both child (ES =  − 0.19) and parent (ES =  − 0.34) reports, with nonsignificant effects for independent evaluator reports. The effects were also small and significant from pre to follow-up for child report (ES =  − 0.36), with insufficient studies available to examine effects for the other reporter types. There were nonsignificant effect differences for remission of depression symptoms according to child and independent evaluator reports from pre to post compared to non-active controls. The effects according to other reporter types could not be evaluated due to small study numbers.

The findings in relation to pre-to-post-treatment effects for reductions in anxiety and depressive symptoms are promising and indicate translation of benefits into routine settings over non-active controls. On initial examination, it appears that the effects from effectiveness trials are smaller than shown in most meta-analyses of efficacy trials particularly for anxiety. For example, reviews of the efficacy of treatments for youth anxiety have indicated average controlled effect sizes of around 0.7 according to reports from either child or parent (James et al., 2020; Reynolds et al., 2012), while reports from youth in trials on the treatment of depression indicate effects of around 0.35 (Eckshtain et al., 2020; Weisz et al., 2017). However, direct comparison is difficult to make since it is confounded with methodology. Of their nature, most studies in routine settings are unable to delay or deny treatment to participants and hence compare their experimental treatment against TAU or another active condition. In fact, over 80% of the trials included in the current meta-analysis compared against TAU, placebo, or an active control. In contrast, the majority of efficacy research delivered through university clinics still utilises waitlist or no treatment controls. Meta-analyses have shown that the effects of treatments when compared against TAU or active controls are considerably smaller than comparison against waitlist (Weisz et al., 2017). For example, in the treatment of youth anxiety, Reynolds et al. (2012) report pre-post effects of 0.76 in comparison to passive controls and 0.35 in comparison to active controls including TAU based on combined effect sizes across reporter. Similarly, in the treatment of youth depression, Eckshtain et al. (2020) report pre-post effects of 0.49 in comparison to no treatment and 0.29 in comparison to TAU based on combined effect sizes across reporter type. In comparison to these figures, the results from the current meta-analysis are not dissimilar. Therefore, it may be that the apparently modest effects from effectiveness trials for youth anxiety and depression is a result of the methods used in these studies (comparison against TAU) rather than any loss of efficacy when moving from university trials to real world application. In line with this, it should be noted that in the studies included in this review, TAU comprised a wide range of interventions including supportive counselling, CBT skills, pharmacology and self-help resources which would be expected to lead to improved symptoms in their own right; hence smaller effect size differences are to be expected.

Direct comparisons with the results of the two previous systematic reviews in routine settings is difficult due to methodological differences. Bear et al.’s (2020) effectiveness review pooled psychotherapy and pharmacotherapy effects, as well as informant type, and included studies in which treatments were delivered by research clinicians making direct comparisons difficult. Wergeland et al. (2021) only included studies of CBT and pooled effects from controlled and uncontrolled studies for within-subject comparisons only. By separating effects for psychotherapy type, informant type and only including controlled studies with evidence for psychotherapy being delivered by routine setting staff, our review provides some unique insights into the effectiveness of psychotherapy in routine settings.

Evidence for longer-term maintenance of outcomes from effectiveness trials over and above non-active controls was limited, particularly in regard to depression outcomes. Overall, there was little indication that the small post-treatment benefits over and above non-active controls lasted into the various follow-up periods. Pre to follow-up effects across child and parent informants were not significantly different from non-active controls for child anxiety but untested for independent evaluator reports. In reporting of depression, children reported significant small (ES =  − 0.36) effect size benefits of psychotherapy over non-active controls at follow-up, with analyses for other informants not conducted due to small sample size. These results indicate ongoing benefits for depression for psychotherapy over non-active controls, but that the benefits of psychotherapy over non-active controls disappears over follow-up periods for anxiety. However, comparisons could not be made for all reporter types due to small study numbers, and independent evaluator reports, in particular, were missing. Hence limited information exists to understand the longer-term effects of interventions over and above non-active controls.

Findings related to remission was exceedingly limited making conclusions difficult. There was evidence according to independent evaluator report of greater remission of anxiety disorders with psychotherapy over non-active controls at post (51.3% vs 36.2%), but not at follow-up (50.9% compared to 38.9% for non-active control), with insufficient data available to examine remission status using child or parent report. This pattern of results matched the results for anxiety symptom severity in which psychotherapy effects over non-active control were no longer significantly greater at follow-up. The lack of difference between therapy condition at follow-up may be due to increased relapse in the psychotherapy condition, increased remission in the non-active control, or a combination of both effects. Regardless of the nonsignificant benefits of psychotherapy over non-active control at follow-up the remission rates remained at relatively high rates. For depression, remission status could only be examined using child (54.1% vs 48.2%) and independent evaluator report (48.6% vs 44.3%) from pre to post, and these comparisons were associated with nonsignificant effects over non-active controls. This finding is perhaps surprising given the significant effects showing benefits of psychotherapy over non-active controls on depressive symptoms from pre to post. However, as not all studies examined remission status, the pattern of findings may relate to the small sample size or other methodological differences between the small number of studies in this analysis. Further the rates of remission in the non-active control were relatively high. Comparisons of remission status at follow-up or for other reporters could not be made. Therefore, whether there are additional benefits of psychotherapy on remission of anxiety and depression over non-active controls is unclear especially at follow-up. The lack of remission data reported in studies is likely due to the challenges of conducting diagnostic interviews in routine settings, especially following discharge from services. It is important to note that while the remission rates for psychotherapy were seldom significantly better than remission rates of the non-active control, the remission rates in both conditions were reasonable with 40–60% of cases remitted. As such, this lends support to the findings noted above that the reason for the smaller effect size benefits of psychotherapy over non-active controls seen in this review likely relate to the relative effectiveness of the non-active control which in the majority of cases was TAU that incorporated known evidence-based strategies. These findings suggest that future reviews need to evaluate TAU components separately.

Most studies were conducted in community settings or schools. Due to small sample sizes, only a few comparisons between setting type could be conducted. Apart from the finding that non-school settings were associated with larger effects than school settings for the treatment of depression according to child follow-up report, all other comparisons indicated no significant differences between setting type and this finding aligns with previous studies that have not found any significant differences in effects across settings (Eckshtain et al., 2020). This favourable outcome suggests that the benefits of psychotherapy for anxiety and depression are apparent across a range of implementation settings and when delivered by a wide range of allied health professionals, medical practitioners, teachers, and trained lay people. As such the benefits of psychotherapy were translatable not only to participants in routine settings, but also when delivered by the variety of professionals who work in these settings.

Across all analyses there was limited evidence that treatment described as CBT was significantly better than other forms of active psychotherapy. Although it is important to note the small sample sizes available, as seven of the studies compared two different types of CBT against each other (e.g. low intensity vs. regular CBT), and therefore could not be included in this analysis. There was one exception where CBT was associated with significant small effects (ES =  − 0.36) for depression with according to child report, from pre to follow-up, but nonsignificant effects from pre to post compared to other therapies. There were insufficient studies to examine differences based on parent or independent evaluator reports for depression. Similarly, anxiety studies did not have sufficient parent report data to examine differences between treatment conditions. The findings related to child and independent evaluator reports showed nonsignificant benefits of CBT over other psychotherapy at pre-post, and at follow-up. On one hand this is perhaps concerning as efficacy studies typically show some benefits of CBT over other psychotherapies (Eckshtain et al., 2020; Reynolds et al., 2012; Weisz et al., 2017), and suggest that CBT may lose its superiority in these settings. However, it is unclear if this effect is due to differences in the population, adherence to CBT protocols, or differences in the differences in the types of active therapies tested. Components of active treatment components was often vague, and delivered in a non-manualised and unmonitored format.

Naturally, the results of the meta-analysis are limited by the quality of the studies within it. Effectiveness trials within routine services are especially difficult to run within the parameters of careful scientific control. As a result, the studies in this review varied widely in methods, measures, and attention to CONSORT guidelines, likely adding to heterogeneity in the results. Therefore, the identified effects need to be interpreted with caution since it is likely that methodological differences and the impact of moderators may heavily influence outcomes. Unfortunately, the relatively small number of studies meant that most obvious moderators such as treatment duration or youth age, were difficult to evaluate. We included studies for the treatment of primary anxiety and mood disorders including PTSD and OCD. The sensitivity analysis indicated that the inclusion of studies for primary PTSD might have influenced the significant outcomes reported for child-reported anxiety and child-reported depression, but not for the other comparisons. It is not known if the inclusion of PTSD studies effected the results due to the increase in sample size, and therefore statistical power for detecting effects, or whether the effectiveness of interventions for PTSD performed differently to those for OCD, anxiety or mood disorders. It should be noted that this finding was only evidence for child-reported symptoms, and so it is unlikely that there were true differences in effectiveness between disorders. It is also important to note that outcomes for primary and comorbid symptoms were examined in combination such that outcomes for secondary disorders not necessarily targeted in treatment were included. This provides a broad understanding of the impacts of interventions, but also might have led to weaker overall effect sizes.

Overall, the results are promising. There were clear benefits of psychotherapy over non-active treatments for anxiety from pre to post across all informants when delivered by setting staff. Similarly, for depression, there were clear benefits of psychotherapy over non-active controls from pre-to-post-outcomes on child and parent report, although the effect for independent evaluator was not significant. Given that the majority of non-active controls were TAU that included evidence-based components this effect might be particularly notable. There was also some suggestion that effects on depression were sustained from pre to follow-up according to child report, with outcomes on the other reporters not tested. Despite the promising findings, more research is needed to understand the true effectiveness of psychotherapy for internalising disorders. Only a small number of studies examined longer-term follow-up or remission. Very few studies compared CBT to another active intervention, and so our understanding of the benefits of different types of psychotherapies in these settings are limited. For anxiety, the benefits of CBT were not significantly different from active control, although the number of studies in the analysis was small and so caution is needed in interpreting the results. For depression, very few studies were available such that comparisons could only be made for child report, which was associated with significant effect (− 0.33) favouring CBT. In conclusion, there is some evidence for the effectiveness of psychotherapy for internalising disorders when delivered in routine settings over non-active controls that predominantly included TAU comparisons, particularly for pre-to post-outcomes, but insufficient evidence for longer-term outcomes, or remission status.

## Supplementary Information

Below is the link to the electronic supplementary material.

Supplementary file1 (DOCX 1616 kb)
