# Supplementary material for: Effectiveness of Psychotherapy for Internalising Symptoms in Children and Adolescents When Delivered in Routine Settings: A Systematic Review and Meta-analysis
Source: Clin Child Fam Psychol Rev. 2023 Apr 14;26(3):824–48. doi: 10.1007/s10567-023-00433-8 (PMC10465434; doi:10.1007/s10567-023-00433-8)
Supplement: Supplementary file 1 — Supplementary file1 (DOCX 1616 kb) [file 10567_2023_433_MOESM1_ESM.docx]

**Appendix A**

**Search Strategies for all Databases**

***PsycINFO***

1. exp Adolescent Psychiatry/ or exp Adolescent Psychotherapy/ or exp Adolescent Psychology/ or exp Adolescent Psychopathology/ or adolescent.mp.

2. exp Child Psychology/ or exp Child Psychopathology/ or child.mp. or exp Child Psychiatry/ or exp Child Psychotherapy/

3. exp Early Adolescence/ or adolescence.mp.

4. exp Community Mental Health Services/ or exp Community Health/ or community care.mp.

5. public health.mp. or exp Public Health/

6. private health.mp.

7. exp Junior High Schools/ or exp High Schools/ or exp Middle Schools/ or school.mp. or exp Schools/ or exp Elementary Schools/

8. major depression.mp. or exp Major Depression/

9. dysthymia.mp. or exp Dysthymic Disorder/

10. affective disorder.mp. or exp Affective Disorders/

11. exp Anxiety Disorders/ or exp Anxiety/ or anxiety.mp.

12. exp Generalized Anxiety Disorder/ or generalised anxiety disorder.mp.

13. exp Separation Anxiety Disorder/ or exp Separation Anxiety/ or separation anxiety disorder.mp.

14. social anxiety disorder.mp. or exp Social Phobia/

15. phobia.mp. or exp Phobias/

16. obsessive compulsive disorder.mp. or exp Obsessive Compulsive Disorder/

17. panic disorder.mp. or exp Panic Disorder/

18. agoraphobia.mp. or exp Agoraphobia/

19. PTSD.mp. or exp Posttraumatic Stress Disorder/

20. adjustment disorder.mp. or exp Adjustment Disorders/

21. intermittent explosive disorder.mp. or exp Explosive Disorder/

22. stress.mp. or exp Academic Stress/ or exp Stress/

23. (cognitive behavior therapy or cognitive behaviour therapy).mp. or exp Behavior Therapy/ or exp Cognitive Behavior Therapy/ or exp Cognitive Therapy/

24. exp Family Therapy/

25. exp "Acceptance and Commitment Therapy"/

26. emotion-focused therapy.mp. or exp Emotion Focused Therapy/

27. psychoanalytic therapy.mp. or exp Psychoanalysis/

28. exp Brief Psychotherapy/ or psychotherapy.mp. or exp Group Psychotherapy/ or exp Psychotherapy/ or exp Individual Psychotherapy/ or exp Interpersonal Psychotherapy/ or exp Psychodynamic Psychotherapy/

29. parent training.mp. or exp Parent Training/

30. e-therapy.mp. or exp Online Therapy/

31. telehealth.mp. or exp Telemedicine/

32. exp Teleconferencing/ or exp Videoconferencing/ or video conferencing.mp.

33. intervention.mp. or exp Intervention/

34. exp Clinical Trials/ or randomised controlled trial.mp. or exp Treatment Effectiveness Evaluation/ or randomized.mp.

35. placebo.mp. or exp Placebo/

36. wait-list control.mp.

37. single-blind.mp.

38. double-blind.mp.

39. exp Treatment Outcomes/

40. social skills training.mp. or exp Social Skills Training/

41. exp School Counseling/ or exp School Based Intervention/ or school counselling.mp.

42. exp Randomized Controlled Trials/ or randomized controlled trial.mp.

43. exp Psychiatric Hospitals/ or exp Hospitals/ or hospital.mp.

44. 1 or 2 or 3

45. 4 or 5 or 6 or 7 or 43

46. 8 or 9 or 10 or 11 or 12 or 13 or 14 or 15 or 16 or 17 or 18 or 19 or 20 or 21 or 22

47. 23 or 24 or 25 or 26 or 27 or 28 or 29 or 30 or 31 or 32 or 33 or 40 or 41

48. 34 or 35 or 36 or 37 or 38 or 39 or 42

49. 44 and 45 and 46 and 47 and 48

***Embase***

1. exp adolescent/ or adolescent.mp.

2. exp child/ or exp child psychology/ or child*.mp. or exp child psychiatry/

3. adolescence.mp. or exp adolescence/

4. exp community/ or exp community care/ or exp community trial/ or community.mp. or exp community mental health center/ or exp community mental health/

5. public health.mp. or exp public health/

6. private health.mp.

7. exp primary school/ or exp high school/ or exp middle school/ or exp school/ or school.mp. or exp school health service/

8. exp major depression/ or exp adolescent depression/ or exp depression/ or exp minor depression/ or depression.mp.

9. dysthymia.mp. or exp dysthymia/

10. anxiety.mp. or exp anxiety/ or exp anxiety disorder/

11. generalised anxiety disorder.mp. or exp generalized anxiety disorder/

12. separation anxiety disorder.mp. or exp separation anxiety/

13. social anxiety disorder.mp. or exp social phobia/

14. exp flying phobia/ or exp spider phobia/ or exp blood-injection-injury phobia/ or exp phobia/ or exp snake phobia/ or phobia.mp. or exp animal phobia/ or exp school phobia/ or exp blood phobia/ or exp needle phobia/ or exp dental phobia/

15. obsessive compulsive disorder.mp. or exp obsessive compulsive disorder/

16. panic disorder.mp. or exp panic/

17. agoraphobia.mp. or exp agoraphobia/

18. PTSD.mp. or exp posttraumatic stress disorder/

19. adjustment disorder.mp. or exp adjustment disorder/

20. exp intermittent explosive disorder/ or intermittent explosive disorder.mp.

21. exp stress/ or exp school stress/ or stress.mp.

22. exp exposure therapy/ or exp cognitive therapy/ or exp family therapy/ or exp behavior therapy/ or exp group therapy/ or exp systemic therapy/ or exp "acceptance and commitment therapy"/ or exp cognitive behavioral therapy/ or exp emotion-focused therapy/

23. psychoanalytic therapy.mp. or exp psychoanalysis/

24. interpersonal therapy.mp.

25. parent training.mp.

26. e-therapy.mp.

27. telehealth.mp. or exp telehealth/

28. video conferencing.mp. or exp videoconferencing/

29. exp randomized controlled trial/ or exp controlled study/ or RCT.mp. or exp controlled clinical trial/ or exp clinical trial/

30. exp placebo/ or exp single blind procedure/ or double-blind.mp. or exp double blind procedure/

31. wait-list control.mp.

32. exp psychotherapy/ or psychotherapy.mp. or exp psychodynamic psychotherapy/

33. exp pediatric hospital/ or hospital.mp. or exp private hospital/ or exp general hospital/ or exp public hospital/ or exp hospital/ or exp community hospital/ or exp mental hospital/

34. 1 or 2 or 3

35. 4 or 5 or 6 or 7 or 33

36. 8 or 9 or 10 or 11 or 12 or 13 or 14 or 15 or 16 or 17 or 18 or 19 or 20 or 21

37. 22 or 23 or 24 or 25 or 26 or 27 or 28 or 32

38. 29 or 30 or 31

39. 34 and 35 and 36 and 37 and 38

***Medline***

1. exp Psychology, Adolescent/ or exp Adolescent/ or exp Adolescent Psychiatry/ or adolescent.mp.

2. exp Psychology, Child/ or child.mp. or exp Child Psychiatry/ or exp Child/

3. adolescence.mp. or exp Adolescent/

4. exp Community Mental Health Services/ or community care.mp. or exp Community Health Services/

5. exp Community Mental Health Centers/

6. public health.mp. or exp Public Health/

7. private health.mp.

8. school.mp. or exp Schools/ or exp School Health Services/ or exp Mental Health Services/

9. hospital.mp. or exp Hospitals/

10. exp Depressive Disorder, Major/ or exp Dysthymic Disorder/ or exp Depressive Disorder/ or exp Mood Disorders/ or exp Depression/

11. exp Anxiety/ or anxiety.mp. or exp Anxiety Disorders/

12. (generalised anxiety disorder or generalized anxiety disorder).mp.

13. separation anxiety disorder.mp. or exp Anxiety, Separation/

14. social anxiety disorder.mp. or exp Phobia, Social/

15. exp Phobic Disorders/ or specific phobia.mp.

16. obsessive compulsive disorder.mp. or exp Obsessive-Compulsive Disorder/

17. panic disorder.mp. or exp Panic Disorder/

18. agoraphobia.mp. or exp Agoraphobia/

19. PTSD.mp. or exp Stress Disorders, Post-Traumatic/

20. adjustment disorder.mp. or exp Adjustment Disorders/

21. intermittent explosive disorder.mp.

22. stress.mp. or exp Stress, Psychological/

23. cognitive therapy.mp. or exp Cognitive Behavioral Therapy/

24. (behavior therapy or behaviour therapy).mp. or exp Behavior Therapy/ [mp=title, abstract, original title, name of substance word, subject heading word, floating sub-heading word, keyword heading word, organism supplementary concept word, protocol supplementary concept word, rare disease supplementary concept word, unique identifier, synonyms]

25. family therapy.mp. or exp Family Therapy/

26. group therapy.mp. or exp Psychotherapy, Group/

27. exp "Acceptance and Commitment Therapy"/ or acceptance commitment therapy.mp.

28. emotion-focused therapy.mp. or exp Psychotherapy/ or exp Emotion-Focused Therapy/

29. psychoanalytic therapy.mp. or exp Psychoanalytic Therapy/

30. interpersonal therapy.mp.

31. parent training.mp.

32. e-therapy.mp.

33. telehealth.mp. or exp Telemedicine/

34. video conferencing.mp. or exp Videoconferencing/

35. (randomized controlled trial or randomised controlled trial).mp. or exp Randomized Controlled Trial/

36. exp Single-Blind Method/

37. exp Double-Blind Method/

38. placebo.mp.

39. wait-list control.mp.

40. active control.mp.

41. 1 or 2 or 3

42. 4 or 5 or 6 or 7 or 8 or 9

43. 10 or 11 or 12 or 13 or 14 or 15 or 16 or 17 or 18 or 19 or 20 or 21 or 22

44. 23 or 24 or 25 or 26 or 27 or 28 or 29 or 30 or 31 or 32 or 33 or 34

45. 35 or 36 or 37 or 38 or 39 or 40

46. 41 and 42 and 43 and 44 and 45

***PubMed***

Search (("adolescent" OR "adolescent/adolescence") OR "adolescence" OR ("adolescent"[MeSH Terms] OR "adolescent/psychology"[MeSH Terms] OR "adolescent behavior/psychology"[MeSH Terms] OR "adolescent psychiatry"[MeSH Terms] OR "adolescent, hospitalized/psychology"[MeSH Terms] OR "adolescent, institutionalized/psychology"[MeSH Terms]) OR ("child"[MeSH Terms] OR "child/psychology"[MeSH Terms]) OR ("child" OR "child/adolescent") OR ("children" OR "children/adolescent")) AND (("community health services/psychology"[MeSH Terms] OR "community health services/therapy"[MeSH Terms] OR "community health workers/psychology"[MeSH Terms] OR "community mental health centers"[MeSH Terms] OR "community mental health services"[MeSH Terms]) OR "community psychiatry"[MeSH Terms] OR ("public health"[MeSH Terms] OR "public health/psychology"[MeSH Terms] OR "public health/therapy"[MeSH Terms]) OR ("school health services"[MeSH Terms] OR "school health services/therapy"[MeSH Terms] OR "school mental health services"[MeSH Terms]) OR "school") AND ("depression" OR ("depression"[MeSH Terms] OR "depression/psychology"[MeSH Terms] OR "depressive disorder"[MeSH Terms] OR "depressive disorder/psychology"[MeSH Terms] OR "depressive disorder, major"[MeSH Terms] OR "depressive disorder, major/psychology"[MeSH Terms]) OR "major depression" OR ("major depressive disorder" OR "major depressive disorders" OR "major depressive episode" OR "major depressive episodes") OR "persistent depressive disorder" OR ("dysthymia" OR "dysthymic" OR "dysthymic disorder" OR "dysthymic disorder/psychology" OR "dysthymic disorders") OR ("dysthymic disorder"[MeSH Terms] OR "dysthymic disorder/psychology"[MeSH Terms) OR ("anxiety"[MeSH Terms] OR "anxiety/diagnosis"[MeSH Terms] OR "anxiety/psychology"[MeSH Terms] OR "anxiety disorders"[MeSH Terms] OR "anxiety disorders/diagnosis"[MeSH Terms] OR "anxiety disorders/psychology"[MeSH Terms]) OR "generalised anxiety disorder" OR "generalized anxiety disorder" OR "separation anxiety disorder" OR ("social anxiety disorder" OR "social anxiety disorder/social phobia") OR ("social phobia" OR "social phobia/social anxiety disorder") OR ("specific phobia" OR "specific phobias") OR ("phobia, social"[MeSH Terms] OR "phobic disorders"[MeSH Terms] OR "phobic disorders/diagnosis"[MeSH Terms] OR "phobic disorders/psychology"[MeSH Terms]) OR "phobia" OR ("obsessive compulsive disorder" OR "obsessive compulsive disorder/psychology") OR ("panic disorder" OR "panic disorder/agoraphobia" OR "panic disorder/psychology") OR ("agoraphobia/panic disorder" OR "agoraphobia/psychology") OR ("posttraumatic stress disorder" OR "posttraumatic stress disorder/ptsd" OR "posttraumatic stress disorder ptsd") OR ("adjustment disorder" OR "adjustment disorder with anxiety" OR "adjustment disorder with anxious mood" OR "adjustment disorder with depressed mood" OR "adjustment disorders" OR "adjustment disorders/psychology") OR "intermittent explosive disorder" OR "stress, psychological"[MeSH Terms]) AND ("therapy, computer assisted/psychology"[MeSH Terms] OR ("cognitive therapy" OR "cognitive therapy ct" OR "cognitive therapy group" OR "cognitive therapy group intervention" OR "cognitive therapy intervention" OR "cognitive therapy interventions") OR ("behaviour therapy" OR "behaviour therapy group" OR "behaviour therapy intervention" OR "behaviour therapy interventions") OR ("behavior therapy" OR "behavior therapy and psychotherapy" OR "behavior therapy based intervention" OR "behavior therapy intervention" OR "behavior therapy interventions") OR ("cognitive behavioural therapy" OR "cognitive behavioural therapy/cbt" OR "cognitive behavioural therapy based intervention" OR "cognitive behavioural therapy based interventions" OR "cognitive behavioural therapy based treatment" OR "cognitive behavioural therapy cbt" OR "cognitive behavioural therapy group" OR "cognitive behavioural therapy group intervention" OR "cognitive behavioural therapy intervention" OR "cognitive behavioural therapy interventions" OR "cognitive behavioural therapy," OR "cognitive behavioural therapy, cbt" OR "cognitive behavioural treatment" OR "cognitive behavioural treatment group" OR "cognitive behavioural treatments") OR ("cognitive behavioral therapy" OR "cognitive behavioral therapy/cbt" OR "cognitive behavioral therapy based intervention" OR "cognitive behavioral therapy based interventions" OR "cognitive behavioral therapy cbt" OR "cognitive behavioral therapy group" OR "cognitive behavioral therapy group intervention" OR "cognitive behavioral therapy intervention" OR "cognitive behavioral therapy interventions") OR ("family therapy" OR "family therapy intervention" OR "family therapy interventions") OR ("group therapy" OR "group therapy intervention" OR "group therapy interventions") OR ("acceptance and commitment therapy" OR "acceptance and mindfulness based interventions") OR ("emotion focused therapy" OR "emotion focused treatment" OR "emotion focused treatments") OR ("psychoanalysis" OR "psychoanalysis/psychodynamic" OR "psychoanalysis/psychotherapy" OR "psychoanalysis and psychodynamic therapies" OR "psychoanalysis and psychology") OR "psychotherapy" OR ("interpersonal therapy" OR "interpersonal therapy group" OR "interpersonal therapy intervention" OR "interpersonal therapy ipt" OR "interpersonal therapy oriented group intervention") OR ("parent training" OR "parent training group" OR "parent training groups" OR "parent training intervention" OR "parent training interventions") OR "e therapy" OR ("telehealth" OR "telehealth/service" OR "telehealth/technology based" OR "telehealth/telecare" OR "telehealth/telemedicine" OR "telehealth/videoconferencing" OR "telehealth/virtual health" OR "telehealth based" OR "telehealth based intervention" OR "telehealth based interventions" OR "telehealth based parent mediated intervention" OR "telehealth behavioral therapy" OR "telehealth delivered") OR ("video conferencing" OR "video conferencing/telehealth" OR "video conferencing based" OR "video conferencing based telemedicine")) AND (("randomised controlled trial" OR "randomised controlled trial design" OR "randomised controlled trial designs" OR "randomised controlled trial rct" OR "randomised controlled trials" OR "randomised controlled trials rct" OR "randomised controlled trials rcts" OR "randomised controlled, crossover" OR "randomised controlled, crossover trial" OR "randomised controlled, double" OR "randomised controlled, double blind" OR "randomised controlled, double blind study" OR "randomised controlled, double blind trial" OR "randomised controlled, parallel" OR "randomised controlled, parallel group" OR "randomised cross" OR "randomised cross over" OR "randomised cross over clinical" OR "randomised cross over clinical trial" OR "randomised cross over design" OR "randomised cross over design study" OR "randomised crossover clinical" OR "randomised crossover clinical trial" OR "randomised crossover trial design" OR "randomised double blind" OR "randomised double blind clinical trial" OR "randomised double blind clinical trials" OR "randomised double blind controlled" OR "randomised double blind controlled clinical" OR "randomised double blind controlled clinical trial" OR "randomised double blind controlled study" OR "randomised double blind controlled trial" OR "randomised double blind controlled trials" OR "randomised double blind cross over design" OR "randomised double blind cross over trial" OR "randomised double blind crossover" OR "randomised double blind crossover trial" OR "randomised double blind design") OR "clinical trial" OR ("controlled clinical trial" OR "controlled clinical trials" OR "controlled clinical trials randomized") OR ("placebo" OR "placebo/control") OR "wait list control" OR ("comparator" OR "comparator control" OR "comparator control group" OR "comparator controlled" OR "comparator controlled clinical studies" OR "comparator controlled clinical trial" OR "comparator controlled clinical trials" OR "comparator controlled phase iii trials" OR "comparator controlled rcts" OR "comparator controlled studies" OR "comparator controlled study" OR "comparator controlled trial" OR "comparator controlled trials") OR "single blind" OR "double blind")

***ERIC***

((adolescent OR adolescence OR children OR child) AND (“community trial" "community care" OR "community mental health” OR public health OR “school mental health”) AND (depression OR “major depressive disorder” OR dysthymia OR anxiety OR “generalised anxiety disorder” OR “generalized anxiety disorder” OR “separation anxiety disorder” OR “social anxiety disorder” OR “social phobia” OR “specific phobia” OR “obsessive compulsive disorder” OR “panic disorder” OR agoraphobia OR “posttraumatic stress disorder” OR “adjustment disorder” OR “intermittent explosive disorder”) AND (“cognitive therapy” OR “behavior therapy” OR “behaviour therapy” OR “cognitive behavior therapy” OR “cognitive behaviour therapy” OR “acceptance commitment therapy” OR “interpersonal therapy” OR psychotherapy) AND (“randomised controlled trial” OR “randomized controlled trial” OR RCT OR placebo OR "wait-list control" OR comparator))

**Appendix B**

**Sensitivity Analysis Testing Inclusion of PTSD**

| **Comparison** | **Primary PTSD included** | **Primary PTSD excluded** |
| --- | --- | --- |
| **Anxiety (child report)** | *d* = -0.26, 95% CI = -0.06, -0.46, Z = 2.51, *p* < .01 | *d* = -0.17, 95% CI = 0.05, -0.40, Z = 1.51, *p* < .13 |
| **Depression (child report)** | *d* = -0.19, 95% CI = -0.02, -0.36, Z = 2.24, *p* < .03 | *d* = -0.17, 95% CI = 0.07, -0.41, Z = 1.39, *p* < .17 |

**Appendix C**

**Subgroup Analyses not Presented in Text**

1. *Psychotherapy vs. Non-Active Controls: Change in Anxiety Symptoms at Post-Treatment by Treatment Setting. Child report.*

1. *Psychotherapy vs. Non-Active Controls: Change in Anxiety Symptoms at Post-Treatment by Treatment Setting. Parent report.*

1. *Outcome 2: Psychotherapy vs non-Active Controls: Remission of Primary anxiety Disorder by Treatment Setting. Independent Evaluator Report.*

1. *Outcome 3: Psychotherapy vs. Non-Active Controls: Change in Depressive Symptoms at Post-Treatment by Treatment Setting. Child Report.*

**

**Appendix D**

**Funnel Plot of Psychotherapy vs. Non-Active Controls Pre-Post on Change in Anxiety Symptoms (Child Report)**

**Appendix E**

**Funnel Plot of Psychotherapy vs. Non-Active Controls Pre-Post on Change in Anxiety Symptoms (Parent Report)**

**Appendix F**

**Funnel Plot of Psychotherapy vs. Non-Active Controls Pre-Post on Remission of Primary Anxiety Disorder (Independent Evaluator Report)**

**Appendix G**

**Funnel Plot of Psychotherapy vs. Non-Active Controls Pre-Post on Change in Depressive Symptoms (Child Report)**
